# Managing the risks and rewards of death in eusocial insects

**DOI:** 10.1098/rstb.2017.0258

**Published:** 2018-07-16

**Authors:** Qian Sun, Kenneth F. Haynes, Xuguo Zhou

**Affiliations:** 1 Department of Entomology, Louisiana State University, Baton Rouge, LA 70803, USA; 2 Department of Entomology, University of Kentucky, S-225 Agricultural Science Centre North, Lexington, KY 40546, USA

**Keywords:** eusociality, undertaking behaviour, necrophoresis, burial, cannibalism, behavioural plasticity

## Abstract

Eusocial insects frequently face death of colony members as a consequence of living in large groups where the success of the colony is not dependent on the fate of the individual. Whereas death of conspecifics commonly triggers aversion in many group-living species due to risk of pathogens, eusocial insects perform cooperative corpse management. The causes and social context of the death, as well as feeding and nesting ecology of the species, influence the way that corpses are treated. The corpse itself releases cues that dictate the colony's response. As a result, social insects exhibit behavioural responses that promote disease resistance, colony defence and nutrient recycling. Corpse management represents a unique adaption that enhances colony success, and is another factor that has enabled eusocial insects to be so successful. In this review, we summarize the causes of death, the sensory detection of death and corpse management strategies of social insects. In addition, we provide insights into the evolution of behavioural response to the dead and the ecological relevance of corpse management.

This article is part of the theme issue ‘Evolutionary thanatology: impacts of the dead on the living in humans and other animals’.

## 1. Introduction

The shift from solitary life to eusociality is one of the major transitions in evolution [1]. Eusociality is rare in the animal kingdom, but the eusocial bees, wasps, ants and termites have achieved extraordinary ecological success and dominate many terrestrial habitats [2]. Eusocial insects typically live in highly complex colonies, which are comparable to human societies on many aspects. They live in densely populated colonies, conduct tasks through division of labour, build complex nesting architectures and engage in extensive social communications [3,4]. One of their intriguing social behaviours is the disposal of dead colony members through removal, burial or cannibalism. This behaviour has fascinated many naturalists and biologists with descriptions of ‘cemeteries’ in early documents, and social insects were once considered the only animals that exhibit this practice other than humans [5–7]. Corpse management has been found both in eusocial hymenopterans (bees, wasps and ants) and in isopterans (termites), and represents a convergent evolution.

Although corpse management in social insects shares similarities with humans in many regards, the underlying mechanisms and the evolutionary significance are different. Social colonies are conceptually analogous to the multicellular organisms and can be considered ‘superorganisms’ [4]. Unlike social practices in human societies where centralized laws and orders are often required and followed, insect societies operate under environmental inputs in a decentralized manner. Tasks are accomplished by individuals through responding to local cues, and social organization emerges via interactions among colony members [8]. In social insects, recognition of death is achieved primarily though olfactory cues, i.e. the post-mortem change of chemical signatures. In humans, the smell of death also triggers threat management responses [9]. The behavioural response in social insects, however, benefits the colony rather than individuals.

Eusociality is characterized by reproductive division of labour with non-reproductive workers, cooperative brood care and overlapping adult generations [3]. This social organization provides many benefits, such as improved foraging efficiency, enhanced defence against predators and increased reproductive success [10]. On the other hand, group living has drawbacks, and one of the major fitness costs is an increased risk of disease. The close genetic relatedness between colony members makes them vulnerable to the same pathogens, and their extensive interactions facilitate the spread of contagious disease [11–14]. Death of the sterile workers or soldiers frequently occurs in insect societies owing to their high density in social colonies. In addition, their lifespans are relatively short compared with the lifespan of the colony, and a high turnover rate of sterile individuals is expected. This is analogous to the high rate of somatic cell turnover throughout the life of an organism [15]. Death not only terminates an individual's contribution to the colony, but also leaves the corpse as a pathogenic risk. Management of corpses is often a prophylactic mechanism to enhance social immunity and represents an essential adaption to social life [12–14].

In 1958, Wilson *et al.* [16] carried out a pioneering study on corpse removal in ants and revealed that decomposition products, fatty acids, are the major death cues. Since then, many studies have been conducted to elucidate the pattern and regulation of this stereotypic behaviour. With a growing interest, researchers have recently discovered novel death cues [17,18], new behavioural patterns and functions [18–21] as well as underlying molecular mechanisms governing death recognition [22,23]. Dead individuals in eusocial colonies, however, represent rewards rather than risks under certain circumstances. Corpse management, which was previously considered as stereotypic, is sophisticated and complex. Social insects often show plastic responses depending on the trade-offs between costs and benefits associated with the nature of corpses, the behavioural strategy employed and the ecology of the species. We start this review by summarizing the causes of death in social insects (§2) and then update current knowledge on the chemical and molecular mechanisms of social response to the dead (§§3 and 4). We also provide an overview of corpse management strategies in eusocial insects compared with non-eusocial species, discuss the costs and benefits of each behaviour, and subsequently introduce behavioural plasticity (§§5 and 6). We hope that this review offers a comprehensive understanding of corpse management in social insects from ecological and evolutionary perspectives, and provides directions for future research.

## 2. Causes of death

**Figure 1.**
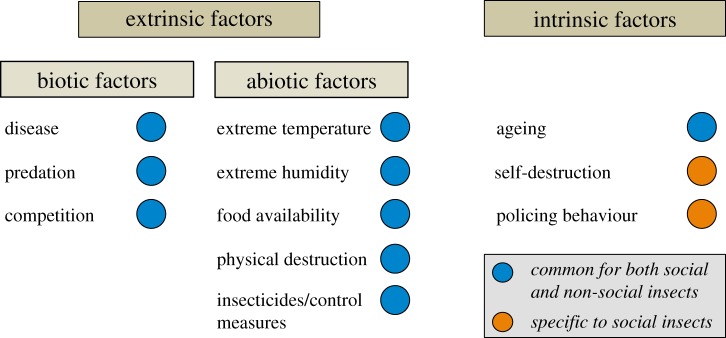
Causes of death in social and non-social insects. In all insects, death commonly results from a variety of extrinsic causes, including both biotic and abiotic factors. In addition, natural ageing eventually leads to death of the individual. In eusocial species, however, two other intrinsic factors contribute exclusively to death, namely self-destruction and policing behaviour.

In nature, the death of insects can result from a variety of causes. Ageing as a natural process is the main cause of death for reproductive individuals, which leads to the death of a colony in many species. Death of sterile individuals (i.e. workers and soldiers), by contrast, can be attributed to other biotic and abiotic factors in addition to ageing (figure 1). Workers are considered as the ‘somatic’ support in the ‘superorganisms’ [24]. Death of workers is a frequent event as they have shorter lifespans [25], and face higher mortality due to taxing and risky tasks such as foraging, colony defence and hygienic activities [26].

Disease is a common cause of death, as the group-living lifestyle and low genetic diversity make social colonies easy targets for pathogenic attacks [11–14]. A wide range of pathogens and parasites (e.g. fungi, bacteria, virus, protozoans and helminths) can infect and kill individuals and spread in the colony. Recent progress in social immunity, the colony-level protection against infectious disease, has revealed the underlying mechanisms and behavioural responses towards infected corpses and dying individuals (reviewed in [13,14]). Moreover, workers and soldiers often die in their defence against predators and competitors. In addition to direct antagonistic interactions, mortality of social insects can also be induced indirectly by the mere presence of predator or competitor cues [27,28]. Common abiotic factors contributing to the death of social insects, as with other insects, include extreme temperature (e.g. freezing and heat), water availability (e.g. desiccation and drowning), lack of food (e.g. starvation) and physical damage to nests by vertebrates or natural disasters. A variety of synthetic pesticides are used in the control of social insects of economic importance, and the behavioural response of colonies to insecticide-killed individuals has been the focus of some applied research [29]. These causes of death, which are associated with different types or levels of risks under natural settings, are expected to influence chemical signatures of dead individuals and elicit different behavioural responses in the living.

While the above-mentioned biotic and abiotic factors can contribute to death in almost all insects, there are two causes of death that occur exclusively in eusocial species, namely self-destructive behaviour in colony defence [30] and policing behaviour to resolve colony conflicts [31] (figure 1). Examples of altruistic self-destruction include the suicidal sting of honeybee workers in their defence against vertebrate intruders [32], old workers of the termite *Neocapritermes taracua* releasing defensive secretions through body rupture [33], workers of the Brazilian ant *Forelius pusillus* sacrificing themselves by routinely closing their nest from the outside to avoid nocturnal predators [34], and sick and dying workers of the ant *Temnothorax unifasciatus* leaving the nest before death to prevent infection of other colony members [35]. While altruism is the foundation of cooperation in eusocial insects, conflicts need to be resolved to maintain colony function. Policing behaviour refers to coercive action that reduces direct reproduction by other individuals. It has been observed widely in social insects in diverse forms, including consuming worker-laid eggs, immobilization, biting and stinging that could eventually lead to the death of focal individuals [36–38]. Self-destruction reflects social strategies to ease risks from natural enemies, and policing behaviour enhances colony efficiency through the regulation of reproductive division of labour.

## 3. Death cues: the novel and conserved chemical signatures

**Figure 2.**
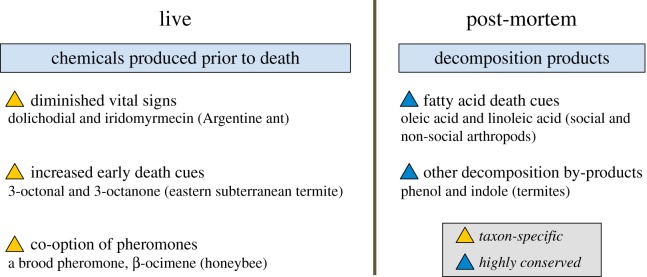
The classification of death cues based on the timing of chemical production. In eusocial species, certain chemicals are produced prior to death but change in quantity or function and, consequently, facilitate early death recognition and elicit corpse management. Examples include dolichodial and iridomyrmecin, which diminish rapidly after death in the Argentine ant, 3-octonal and 3-octanone, which increase immediately upon death in the eastern subterranean termite, and a brood pheromone (β-ocimene), which recruits workers for brood removal in the honeybee. These chemicals are probably taxon-specific and represent evolutionary novelties in eusocial insects. Decomposition products are produced post-mortem. Fatty acids are highly conserved death recognition cues both for social and for non-social arthropods. Phenol and indole are probably conserved volatile death cues in termites.

Individuals that die within a colony must be recognized by colony members for efficient and timely management. Recognition of dead individuals and elicitation of corpse management are primarily achieved by olfactory cues. Death cues, here, refer to post-mortem changes of surface chemicals that mediate behavioural responses in live insects. Depending on the timing of chemical production (before or after death), we classify them into two categories, ‘chemicals produced prior to death’ and ‘chemicals produced post-mortem’, i.e. ‘decomposition by-products' (figure 2).

The first category includes chemicals actively synthesized in live insects, which change in quantity or function after death. For instance, in the Argentine ant *Linepithema humile*, dolichodial and iridomyrmecin are produced in the pygidial gland and present on the cuticle of live workers to mask the corpse removal stimuli, triglycerides. The rapid decline of the two compounds after death (within 40 min) allows workers to recognize death and remove the corpses before substantial decomposition occurs [17]. In the eastern subterranean termite, *Reticulitermes flavipes*, workers release two volatiles, 3-octonal and 3-octanone, synchronously with death, stimulating nest-mates to detect and locate the corpses [18]. These two volatiles are not released, although presumably produced and stored, in live workers. After death, they rapidly peak in quantity to recruit undertakers in a timely manner and then gradually decrease. In the honeybee *Apis mellifera*, a volatile brood pheromone, β-ocimene, which signals food begging, continues to emit after death and recruits workers to uncap and remove dead larvae [23]. The above-mentioned examples represent three distinct patterns of using chemicals produced prior to death for the timely detection of death: diminished vital signs, increased early death cues and co-option of pheromones, respectively.

The second category of death cues, decomposition by-products, refers to chemicals produced after death. In a pioneering study of chemical stimuli for corpse disposal, Wilson *et al.* [16] found that oleic acid was the most effective stimulus eliciting corpse removal in two ant species, *Pogonomyrmex badius* and *Solenopsis saevissima*. Since then, ‘fatty acid death cues’ have been found in many other social species, including the archaic ant *Myrmecia vindex* [39], the red ant *Myrmica rubra* [20], the fire ant *Solenopsis invicta* [40], a fungus-growing termite *Pseudacanthotermes spiniger* [41], two subterranean termites *Reticulitermes virginicus* [42] and *R. flavipes* [18], as well as the honeybee *A. mellifera* [23]. Oleic acid and linoleic acid are the most common stimuli for corpse disposal in those species; the two compounds have low volatilities and require direct contact or short distance detection. Phenol and indole, which are decomposition products of proteins, are additional volatile death cues involved in corpse burial in the termites *P. spiniger* and *R. flavipes* [18,41]. While volatile death cues facilitate the orientation of insects from a distance, low-volatility cues, such as fatty acids, allow workers to pinpoint the specific location of the dead individual that requires disposal [43]. Fatty acids accumulate in dead individuals as a result of autolytic catabolism or bacterial hydrolysis of triglycerides [44]. The ‘fatty acid death cues' represent a group of conserved post-mortem chemical signatures that trigger avoidance in gregarious species in both Crustacea and Hexapoda, including amphipods (Amphipoda) [45], woodlice and pill bugs (Isopoda) [46], springtails (Collembola) [47,48], crickets (Orthoptera) [49], cockroaches (Blattodea) [46,50], booklice (Psocoptera) [51] and social caterpillars (Lepidoptera) [46]. These compounds are comparable to cadaverine and putrescine in vertebrates, which are emitted from decaying flesh and stimulate burial in rats [52] and aversion in fish [53].

While decomposition products usually require longer post-mortem time to accumulate and affect behaviour, chemicals produced prior to death facilitate early detection and timely prophylactic corpse management. These early death cues, although emanating from dead individuals, function similarly to ‘pheromones’. By contrast to the decomposition by-products, which are taxonomically conserved death cues, the chemicals produced prior to death are probably specific to species or closely related lineages (e.g. genus). We propose that the use of chemicals produced prior to death represents evolutionary novelties and that it occurs widely in social species, where early detection and disposal of corpses enhances colony fitness.

In contrast to olfactory cues, no evidence supports a role for visual, auditory or thermal cues for death recognition in social species examined so far. However, possible roles for such cues cannot be excluded, owing to the limited number of species investigated, available toolsets and the predominant focus on chemical cues in most studies. Tactile cues (e.g. glass beads carrying candidate chemicals) were found to synergize the effect of oleic acid on burial behaviour in a termite *R. virginicus* [42], suggesting potential non-olfactory contributions to corpse management. In most studies focused on death cues, freezing has been used as a standard protocol to induce death. A few chemical cues from diseased (and dying) brood that trigger hygienic behaviour have been identified in honeybees [54,55]. In the ant *Lasius neglectus*, fungal infection alters the cuticular hydrocarbon profile of pupae, which triggers workers to kill both infected individuals and the pathogen (i.e. destructive disinfection) [43]. Although a variety of other factors can contribute to the death of social insects, olfactory cues associated with different causes of death are largely unknown.

## 4. Perception of death cues and molecular basis of corpse management

Olfaction is the key sense for insects to detect their chemical environment [56]. In social insects, odorant-binding proteins (OBPs), odorant receptors and other chemosensory proteins are expected to be involved in the perception of diverse death cues. In the fire ant *S. invicta*, a chemosensory protein gene, *Si-CSP1*, was found to be responsible for detecting oleic acid and linoleic acid and thus mediating corpse removal [22]. In the honeybee *A. mellifera*, both oleic acid and β-ocimene are ligands for two OBPs (OBP16 and OBP18), which are associated with hygienic behaviour (removal of diseased brood) [23,57]. Receptors for either conserved fatty acid death cues or species/taxon-specific death cues remain unknown in insects. In addition to genes encoding proteins for death cue perception, others are expected to influence the behavioural response, as corpse management is a complex and sequential behaviour involving multiple steps and often dependent on the social context (see §§5 and 6).

Pheromones and other social stimuli can influence gene expression, which further affects behaviour or physiology of the recipients [58–61]. Comparison of gene expression patterns between individuals that specialize in corpse management and that do not, and before and after exposure to death cues, can be informative to determine the genetic underpinnings of corpse management. This approach has been applied to analyse gene expression associated with hygienic behaviour in the honeybee [62–64]. Gene expression in the brain of undertaker bees, which are temporarily specialized in corpse removal, is similar to that of guards but slightly different from comb builders [65]. Our recent transcriptome analysis in the eastern subterranean termite *R. flavipes* found that death cues cause changes in gene expression in workers within 30 min, and different sets of genes are associated with corpse cannibalism versus burial (unpublished data). Although our understanding of corpse management at the molecular level is in its infancy, the advent of genomics and functional genomics technologies, including the next-generation sequencing, RNAi and genome editing, will facilitate our efforts in the near future.

Remarkable progress has been made in the field of sociogenomics, unveiling the molecular basis of sociality from altruistic behaviour to division of labour [66–68]. One emerging theme is that genes involved in simple, non-social behaviour can be used for complex social behaviour. For example, the *foraging* (*for*) gene, encoding a highly conserved cGMP-dependent protein kinase, can cause a sitter to display rover-like behaviour in *Drosophila* [69] and facilitate the transition of gregarious desert locusts, *Schistocerca gregaria*, into solitary ones [70]. In eusocial insects, *for* functions as a positive regulator for foraging behaviour in the honeybee, *A. mellifera* [71], and a negative regulator in the bumblebee, *Bombus ignites* [72], the common wasp, *Vespula vulgaris* [73], the harvester ant, *Pogonomyrmex barbatus* [74,75], and the termite, *R. flavipes* (unpublished data). The detection and recognition of fatty acids as death cues is prevalent in arthropods, suggesting conserved genes/gene networks in the receiving end (perception) of these compounds. With readily available genomes, the molecular dissection of oleic acid perception and the downstream signalling pathway in the social hymenopterans might be the most logical initial steps in investigating corpse management at the mechanistic level. In the meantime, an array of taxa should be analysed to understand how corpse management has evolved from a simple ancestral trait of death recognition to a complex sequential behavioural response.

## 5. Behavioural responses: a comparison between social and non-social species

The behavioural response to corpses, also called ‘undertaking behaviour’, has evolved into diverse forms in eusocial insects, including corpse removal, cannibalism and burial. The strategies of undertaking behaviour depend on the feeding and nesting ecology of the species. Even within the same species, undertaking response to the dead can vary because of the risks and rewards associated with the corpses. The behavioural strategies employed in different groups of social insects and detailed behavioural responses have been previously reviewed [7,76]. In this review, we compare similar behaviours in non-eusocial species with corpse management in eusocial species, and discuss the potential costs and benefits of each behavioural strategy, as well as the ecological relevance of corpse management (table 1).

**Table 1.** Corpse management in eusocial insects. NA, not available, which indicates that the behaviour is absent or not reported in the given eusocial group.

behavioural strategy	description	terminology	nesting and feeding habits	costs	benefits	eusocial insects	non-eusocial arthropods^a^
avoidance	intentionally staying away from the dead or where corpses are located	necrophobia	non-permanent nest, or flexible nest structure	losing at least partially the nest; increased labour and energy input in nest reconstruction or relocation; risk of predation during relocation	preventing disease transmission in colony	wasps (NA), ants [77–79], bees [80,81], termites [29,82–84]	amphipods [45], woodlice and pill bugs [46], springtails [47,48], crickets [49], cockroaches [46,50], booklice [51], caterpillars [46]
corpse removal	carrying the dead out of nest, to refuse piles or specialized chambers; leaving the nest before death (self-removal)	necrophoresis	enclosed nest	risk of contagion at individual level; risk of predation at individual level; reducing individual lifespan in the case of self-removal	preventing/reducing disease transmission in colony; saving nest space	wasps [85], ants [7,16,17,20,39,40,76,86,87], bees [7,88,89], termites (NA), aphids [90]	*waste removal*: spiders [91], spider mites [92], webspinners [93], crickets and grasshoppers [94,95], cockroaches [96], bark and ambrosia beetles [97]
cannibalism	consuming dead, dying or injured conspecific individuals	intraspecific necrophagy	nutrition-imbalanced food or seasonal food shortage	risk of contagion at individual level	preventing/reducing disease transmission in colony; recycling nutrients and potentially symbionts	wasps (NA), ants [86,98–103], bees (NA), termites [7,18,76,84,104–106]	*cannibalism of live individuals*: shrimps [107], spiders [108], mantids [109], crickets [110], cockroaches [111], assassin bugs [112], fruit fly larvae [113], lady beetles [114], moths [115]
burial	covering dead individuals or blocking the areas where corpses are present with soil and/or other materials	entombment	enclosed nest	risk of contagion at individual level; labour and energy intensive	preventing/reducing disease transmission in colony; colony defence against predators or competitors	wasps (NA), ants [19,39,116], bees [117–119], termites [18,41,83,84,106,120,121]	*burying dead brood*: ambrosia beetles [122]; *burying carrion as food source*: burying beetles [123]

^a^Similar behaviours in non-eusocial arthropods are summarized.

### (a) Avoidance

Avoiding the dead, also known as necrophobic behaviour, is considered a behavioural mechanism to manage threats such as predation and disease. Avoidance of dead individuals or smells associated with death is common in animals ranging from arthropods to fishes, birds and mammals, including primates [46,53,124–127]. Among arthropods, avoiding dead conspecifics has been found in a wide range of non-eusocial but gregarious species [45–51]. While staying away from the dead is an effective solution to manage risks in these species, it is not commonly observed in eusocial species that live in permanent nests. Avoiding dead individuals in the nest means eventually abandoning the nest. Relocating or reconstructing the nest requires labour input from the entire colony and may pose increased risk of predation to the brood and reproductive individuals. Social insects only employ this strategy when the level of risk from not doing so is not manageable. For example, the fire ant *S. invicta* opts to relocate the nest only when it is heavily infected with nematodes or fungal pathogens [77,78]. In species that live in simple nests or frequently relocate, such as the rock ant, *Temnothorax albipennis*, workers avoid new nest sites containing conspecifics’ corpses [79] (but see also contrary behaviour in the pharaoh ant, *Monomorium pharaonis* [128]). In subterranean termites, the nest is a complex structure composed of dynamic foraging galleries and chambers expanding to thousands of square metres. The colony size can reach millions of individuals [129]; therefore, the costs of relocating the entire colony are prohibitive. Workers avoid dead individuals infected with pathogens or killed by insecticides by sealing off (i.e. burying) the area where corpses are located [29,82,83]. In other words, subterranean termites simply modify the nesting structure locally, rather than relocating, indicating that avoidance in termites is less energetically costly than in other species. Avoidance of corpses often occurs following burial or construction to prevent contagion, but avoidance without burial activity is also observed in a higher termite *Globitermes sulphureus* under laboratory conditions [84].

### (b) Corpse removal

Corpse removal, also called necrophoresis, was coined initially by Wilson *et al*. [16] to describe social insects carrying dead colony members away from the nest. Although behavioural processes are similar, corpse removal is a derived social behaviour distinguishable from waste disposal, in that corpses are removed in a timely manner and dropped further away from the nest [16,17,88]. Management of waste materials, such as faeces and food remains, is a sanitation practice in eusocial insects [130–132], as well as many subsocial species including spiders [91], spider mites [92], webspinners [93], crickets and grasshoppers [94,95], cockroaches [96], bark and ambrosia beetles [97]. In the eusocial gall-forming aphids, *Pemphigus spyrothecae*, soldiers dispose of corpses in the same manner as nest wastes [90], suggesting that they may not distinguish death cues from other aversive odours, or that dead nest-mates and other wastes both pose pathogenic risk. Based on current knowledge, we predict that corpse removal has evolved from waste removal. This hypothesis can be tested by examining the olfactory and behavioural response towards corpses and other wastes in eusocial and closely related non-eusocial species within the same lineage.

Corpse removal is the most common management strategy in ants and bees [16,17,20,39,40,86–89] (and see summaries in reviews [7,76]). One worker can carry one corpse at a time; therefore, it is not a labour-intensive behaviour, and it is expected to be an efficient solution when the number of corpses is low. This behaviour provides fitness benefits to the colony through keeping the nest a sanitary environment [133]. Workers that perform this behaviour, however, may expose themselves to risks of infection or predation if the corpse is carried outside the nest.

Stereotypic corpse removal has been found only in eusocial insects, not subsocial or communal species, implying that it is a consequence of the evolution of eusociality. Their highly complex social living leads to increased frequency of death inside the nest and increased risks of pathogen transmission from the dead, and corpse removal is a behaviour performed primarily by workers [12,13]. Besides sociality, nesting ecology is the other important factor in shaping the behaviour. Removal of dead individuals is not expected in species living in open nests where dying inside the colony is less likely, such as paper wasps. However, removal of dead or diseased brood (i.e. hygienic behaviour) is common in both wasps and bees, because the immature stages are reared in confined cells [85,134]. In addition to hygienic benefits, removal of dead or dying brood also allows the colony to re-use the nest space. In an extreme case, when removing individual brood is not sufficient to eliminate infestation of phorid flies, the queen of the social wasp, *Mischocyttarus labiatus*, cuts off the entire comb to remove all brood, and constructs a new comb with the help of her workers [85], representing a highly costly behaviour similar to avoidance to negate high risk.

It is interesting that some ants leave the nest when they are dying owing to certain pathogen infections [35,135]. Self-removal has been explained as parasitic manipulation of host behaviour [136,137], but non-manipulating generalist fungi, such as *Metarhizium brunneum*, can also elicit this behaviour. A recent study in *M. rubra* found that dying workers, infected with *Metarhizium brunneum*, left the nest due to impaired olfactory function [138]. However, this behaviour also represents a form of altruism, because non-infected individuals are also found to withdraw from the nest when they are moribund, such as in *T. unifasciatus* [35]. Dying away from the colony can limit disease transmission through a less costly approach because it requires no additional input from the colony, but the lifespan of the worker that performs the behaviour is reduced [35]. Self-removed corpses are scattered outside the nest and can be re-encountered by other ants, and, as a result, transmit disease to their nest-mates. We predict that self-removal evolves only in species with small colonies or small nesting ranges, whereas in ants that live in large colonies or range across large areas, carrying corpses to refuse piles is more effective to reduce re-encountering pathogen sources.

### (c) Cannibalism

Cannibalism describes the consumption of conspecifics; it is not specific to eusocial insects. Cannibalism of live individuals is widespread in animals, including predatory cannibalism [110,113], sexual cannibalism [108,109] and brood cannibalism [112]; it rewards the cannibals with nutrients and energy [109,110]. Eusocial insects also cannibalize live brood or other colony members under stressful conditions such as starvation, to regulate resources [139,140], or consume worker-laid eggs to resolve reproductive conflict [141]. Here, cannibalism that qualifies as corpse management practices is also called intraspecific necrophagy, which refers to the consumption of dead, dying or injured conspecifics. Cannibalism provides nutritional benefits as in other species, and in social species it also benefits the colony by eliminating the potential source of pathogens. However, cannibalism has also been considered to increase the risk of pathogen uptake by the cannibals [142,143].

Cannibalizing the dead is rare in ants (but see [86,98–102]), but can happen during seasonal food shortages, indicating that dead conspecifics can be used as a food supply [103]. Consumption of dead nest-mates has not yet been reported in wasps or bees. In termites, however, corpse cannibalism has been documented in diverse species [18,84,104–106] (see also summaries in previously published reviews [7,76]). Termites primarily feed on wood, which is rich in carbon but poor in nitrogen, and cannibalism of corpses is an important mechanism for nitrogen recycling [144]. In two higher termite species, *Microcerotermes crassus* and *G. sulphureus*, which feed on highly decomposed plant materials with higher nitrogen content, cannibalism of the dead rarely occurs, supporting the role of feeding habit in corpse management [84]. Termites rely on a variety of gut symbionts to digest lignocellulose, and these symbionts are transferred among nest-mates through proctodeal trophallaxis and coprophagy [145]. Cannibalism of newly dead individuals potentially allows termites to acquire symbionts [146], but the hypothesis of symbiont recycling requires further testing. Cannibalism in termites is restricted to freshly dead and dying individuals [18,84,147], thus reducing the loss of nutrients (and possibly symbionts) and minimizing the risk of disease transmission due to pathogen development during decomposition. The risk of infection by cannibalism can be mitigated through antimicrobial properties in termite saliva and guts [148–150].

### (d) Burial

Although burying the dead with soil or other materials is not common in non-eusocial species, it can be found in ambrosia beetles, in which females bury the dead and weak brood [122]. Burying beetles are known to bury small vertebrate carrion as a food source for their larvae; this is a parental care behaviour that serves a different function from corpse management [123].

Ants generally prefer corpse removal, and corpse burial is an uncommon behaviour with only a few cases reported. For example, *M. vindex* buries objects treated with oleic acid [39], *Temnothorax lichtensteini* tends to bury freshly dead corpses of a foreign species [19] and *S. invicta* covers fungus-infected corpses with soil in artificial nests, which reduces transmission of the disease [116]. In the black garden ant, *Lasius niger*, co-founding queens bite and bury dead co-foundresses in closed nests where removal is impossible, and such undertaking behaviours improve their survivorship [151]. Although honeybee workers rarely practice burial behaviour, they use propolis (plant-produced resins used in the hive) to entomb dead mice or large insects that are not removable [117–119], and to encapsulate nest intruders such as parasitic beetles [152]. In termites, although cannibalism brings nutritional benefits, burial is more efficient when corpses are in large number, as cannibalism takes a longer time and requires more workers [106]. Termites also bury corpses that are highly decomposed [18,84], highly infected [106,120], killed by insecticides [29,83] or from competitor species [121,153].

Taken together, these observations suggest that burial behaviour is preferred when corpses pose higher risks or other behavioural strategies are impractical. Infected corpses indicate direct risk of pathogenic attack, and those that are highly decomposed or in large quantity also suggest increased pathogenic risks. Corpses from a foreign species imply predatory or competitive risks, or risks of unknown pathogens that other disease defensive mechanisms in the focal species may not cope with [19,121]. Compared with corpse removal, burial behaviour is costly. Burial is a collective behaviour that requires more labour force and energy than removal [18], and it often involves utilization of antimicrobial compounds secreted in saliva or excreted in faeces [154]. However, burial seems to be the most effective behaviour to suppress disease transmission in the nest, as it prevents any further contact and decreases the decomposition process through physical isolation [155]. In addition, burial functions as a defensive mechanism against potential intruders, as it blocks the entrance where more intruders may be present, thus preventing further aggression [153].

## 6. Behavioural plasticity to manage risks and rewards

Corpses pose different types or levels of risks and rewards according to their nature, such as post-mortem time, cause of death, origin and quantity. These characters can be recognized via different death cues, and they elicit differential responses in social insects. Furthermore, the social context and other environmental conditions provide additional information, which social insects integrate with death cues to evaluate the risks and rewards, and make the management decision.

### (a) Differential response influenced by the nature of corpses

Corpses decompose over time, and surface chemicals change with post-mortem time. Honeybees and ants can distinguish dead nest-mates with different decomposition status, and remove those that have decomposed for longer more rapidly [20,88]. In *M. rubra*, this process is dictated by the level of fatty acids that accumulate after death [20]. In termites, dead and injured individuals offer nutritional rewards, but the nutritional value drops and the risk of pathogenic attack increases as the corpse decomposes. The trade-off between nutritional rewards and pathogenic risks leads to a behavioural shift from cannibalism to burial in *R. flavipes*, *R. speratus* and *Coptotermes formosanus* [18,84]. In *R. flavipes*, this behavioural plasticity is regulated by the dynamic change of death cues over time, which include an early death cue composed of two volatiles that recruit workers to locate and consume the dead and late death cues composed of mainly fatty acids that trigger burial [18]. The behavioural regulation of risks and rewards associated with corpses is comparable to the care–kill dichotomy in social immunity, which refers to the differential behaviour towards diseased colony members according to whether the individual can be cured or poses a threat to colony fitness [14,43,147].

Corpses that die from disease pose a direct risk of epidemic outbreak in the colony; therefore, rapid behavioural response or more effective strategies are expected. For instance, in the fire ant *S. invicta*, dead pupae infected with a fungal pathogen *Metarhizium anisopliae* are removed to the refuse pile more promptly than non-infected pupae [40]. When the same fungal pathogen kills individuals in the subterranean termite *R. virginicus*, workers bury the diseased corpses whereas they cannibalize pathogen-free corpses [105].

Social insects distinguish nest-mates from non-nest-mates and recognize castes within a colony through cuticular hydrocarbons (CHCs) [156], which remain on the surface of the individual after death for a period of time [20]. CHCs, therefore, can provide information about the identity of corpses and influence behavioural response. Non-nest-mate corpses, representing additional threats such as competition, predation and foreign pathogens, elicit complex behaviour different from nest-mate corpses. For example, in *R. flavipes*, freshly dead individuals from a competitor species, *R. virginicus*, trigger intensive burial behaviour in workers while soldiers are recruited to guard the burial site and attack the dead [121]. Similarly, in the ant *T. lichtensteini*, workers bury and bite newly dead alien corpses, whereas they normally remove dead nest-mates [19]. And in *M. rubra*, freshly dead alien corpses are removed more frequently and elicit more aggression than nest-mate corpses [20]. Interestingly, in *Formica cinerea*, corpses from a territorial competitor and a slave-maker species provoke aggression and are quickly carried inside the nest rather than outside, which is presumably a behavioural mechanism to avoid further detection by live intruders [21]. Developmental stages of corpses also influence the behavioural response. In a bumblebee, *Bombus terrestris*, workers remove larval corpses faster than adult corpses [157], while in the ant, *S. invicta*, dead pupae are removed more slowly than dead workers [40].

### (b) Differential response influenced by social context

Social insects often display differential behaviour in a context-dependent manner, even towards the same stimuli. For example, in the ant *Temnothorax rugatulus*, alarm pheromone repels or attracts nest-mates depending on whether it is released in an unfamiliar site or in the vicinity of the nest [158]. In the dampwood termite, *Hodotermopsis sjostedti*, workers show increased aggression towards intruders in the presence of reproductive caste, but reduce aggressive activities when soldiers are present [159]. Social context provides important information regarding risks and rewards, and social insects are remarkably flexible in their behavioural response. This is also true in corpse management practices. For example, bees and ants may encounter dead conspecifics during foraging, but only those that die inside or near the nest present risks to the colony and trigger corpse disposal. Bumblebees and honeybees are known to remove corpses from the nest, but they show avoidance when foraging on flowers with death cues of conspecifics [80,81]. This suggests that the context of ‘nest’, presumably recognized by chemical cues or physical properties, is associated with risks or rewards at colony level and thus a prerequisite for corpse management. Fatty acids are common death cues initiating corpse management; however, they also appear on food sources such as dead insects or seeds that many ants feed on [160]. Although synergistic chemical cues possibly allow social insects to discriminate food from dead colony members, social context plays a role in their response. In the ant *Pogonomyrmex badius*, oleic acid elicits necrophoresis when most of the ants are engaging in nest maintenance or cleaning, but induces foraging behaviour if the colony is actively feeding or convening [161]. Another example concerns behavioural plasticity in the reproductive caste. Queens do not perform non-reproductive activities in mature colonies, but in newly founded colonies where worker helpers are not available, they engage in tasks of corpse disposal. This has been documented in a fungus-growing termite, *P. spiniger*, and an ant, *L. niger* [41,151]. In *L. niger*, biting and burying dead co-foundresses by queens when removal from closed nests is restricted [151] illustrates how nest structure and environmental conditions can play a role in behavioural plasticity. When disposing of corpses outside is restricted due to factors such as nest blockage, flooding or freezing, alternative behaviours such as burial or cannibalism are expected. In addition, colony size influences the behavioural response in the management of infection. When challenged with objects bearing fungus spores, *M. rubra* workers living in large colonies removed the infected items fast, whereas workers in small colonies relocated themselves and larvae first, and returned to the nest after waste items were removed by a few individuals [162].

## 7. Conclusion

Death in social colonies occurs due to various factors, which in turn present different risks and rewards to the colony. Death cues differ according to the nature of the corpses and change over time. To manage death properly, social insects must discriminate between the dead and the alive, distinguish corpses of nest-mates from non-nest-mates, and locate and assess the status of corpses. With all the information integrated, they perform a specific behaviour, such as corpse removal, cannibalism, burial or avoidance. All of these behavioural mechanisms serve the function of disease resistance, as one of the major threats posed by dead individuals is pathogen transmission. However, corpse management is not only a hygienic behaviour, but also benefits the colony through nutrient recycling and promoting defence against intruders. In addition, the nesting structure and feeding habits of a given species are important factors in evaluating the risks and rewards associated with corpse management strategies.

Most studies have focused on behavioural analyses and the chemical bases of death cues. The causes of death at individual and colony levels, which provide critical information for understanding the chemical cues and behavioural responses, have not been thoroughly investigated. Few species have been investigated, with a bias towards social insects of economic importance, such as invasive ants (e.g. fire ant and Argentine ant), honeybees and subterranean termites. Corpse management in wasps, ants that live in smaller and simpler societies, and primitive species of termites (such as drywood and dampwood termites) remain mostly unexplored. Many analogous behavioural responses can be found in non-eusocial species, which can be helpful in determining how specific behavioural responses were shaped during the evolution of eusociality. In addition, evaluation of the benefits of corpse management at colony level, which provides proxies for fitness advantages, has so far been studied in only one species [133]. To better understand how corpse management has evolved in different social groups, we look forward to studies on comparative analyses of costs and benefits between behavioural strategies, direct measurement of fitness value associated with corpse management, and phylogenetic analyses of eusocial and non-eusocial species exhibiting similar behaviour with consideration of their nest and feeding ecology.

## Data accessibility

This article has no additional data.
